# An Unexpected Culprit: Nocardia Pericarditis Leading to Cardiac Tamponade in an Immunocompetent Host With Congenital Heart Disease

**DOI:** 10.7759/cureus.104519

**Published:** 2026-03-01

**Authors:** Devendra K Jain, Sandeep Seth, Gagandeep Singh, Angitha K Parambath, Shitij Chaudhary

**Affiliations:** 1 Cardiology, All India Institute of Medical Sciences, New Delhi, New Delhi, IND; 2 Medical Microbiology, All India Institute of Medical Sciences, New Delhi, New Delhi, IND; 3 Cardiology, Dayanand Medical College and Hospital, Ludhiana, IND

**Keywords:** atrial septal defect, cardiac tamponade, nocardia, pericardial effusion, trimethoprim-sulfomethoxazole, tuberculosis

## Abstract

We report the case of a woman in her 40s with type II diabetes mellitus and a known ostium secundum atrial septal defect (23 mm, left-to-right shunt) diagnosed one year back. The patient presented with progressive dyspnea on exertion for the last three months, along with low-grade fever and fatigue. Clinical and echocardiographic evaluation revealed massive pericardial effusion with features of cardiac tamponade. Pericardial drainage via a pigtail catheter yielded 650 mL of exudative fluid. Pericardial effusion was negative for fungal culture, malignant cytology, and tubercular workup. Adenosine deaminase was negative, and the effusion was predominantly neutrophilic and exudative in nature. The bacterial culture showed the presence of *Nocardia*. The patient was started on trimethoprim-sulfamethoxazole, following which there was significant improvement in clinical status over the next month, and the pericardial effusion resolved. This case highlights the importance of early microbiological diagnosis and targeted antimicrobial therapy in patients with pericardial effusion to optimize clinical outcomes, particularly in tuberculosis-endemic countries, where exudative pericardial effusion is often presumed to be of tubercular origin.

## Introduction

Pericardial effusion and cardiac tamponade are potentially life-threatening complications that can occur secondary to a wide spectrum of etiologies, including infections, malignancy, autoimmune disorders, and metabolic causes. Among the infectious etiologies, *Mycobacterium tuberculosis* remains a leading cause of pericarditis in developing countries such as India [1]. Due to a lack of diagnostic facilities, very often, pericardial effusion is attributed to tuberculosis due to its high prevalence.

However, *Nocardia* species, filamentous, gram-positive and weakly acid-fast aerobic bacteria, represent an exceedingly rare cause of pericardial infection, typically occurring in immunocompromised hosts, such as those with human immunodeficiency virus (HIV) infection, organ transplantation, or long-term corticosteroid use [2]. Due to the similarities in staining characteristics (partially acid-fast) and morphology with *Mycobacterium tuberculosis*, nocardiosis is frequently misdiagnosed as tuberculosis [3]. In immunocompetent individuals, nocardial pericarditis is exceptionally uncommon, with only isolated cases described in the literature. The clinical presentation often mimics tuberculous pericarditis, leading to diagnostic delays and inappropriate therapy [3]. Microbiological confirmation through prolonged culture and modified Ziehl-Neelsen staining remains the cornerstone of diagnosis [2].

We report a rare case of massive pericardial effusion in a middle-aged woman with a background of atrial septal defect (ASD), where *Nocardia* species was isolated from pericardial fluid. The patient was immunocompetent and initially treated empirically for tuberculous pericarditis. This case highlights the importance of maintaining a high index of suspicion for atypical organisms such as *Nocardia*, even in immunocompetent hosts, especially in endemic settings where tuberculosis is the default diagnosis for exudative pericardial effusions.

## Case presentation

A woman in her 40s, a known case of type II diabetes mellitus and congenital heart disease with ostium secundum ASD (23 mm in size, with left-to-right shunt), presented with progressive dyspnea over one year, worsening during the preceding three months, associated with low-grade fever and fatigue for two weeks. There was no prior history of tuberculosis or immunosuppressive drug use.

**Figure 1:**
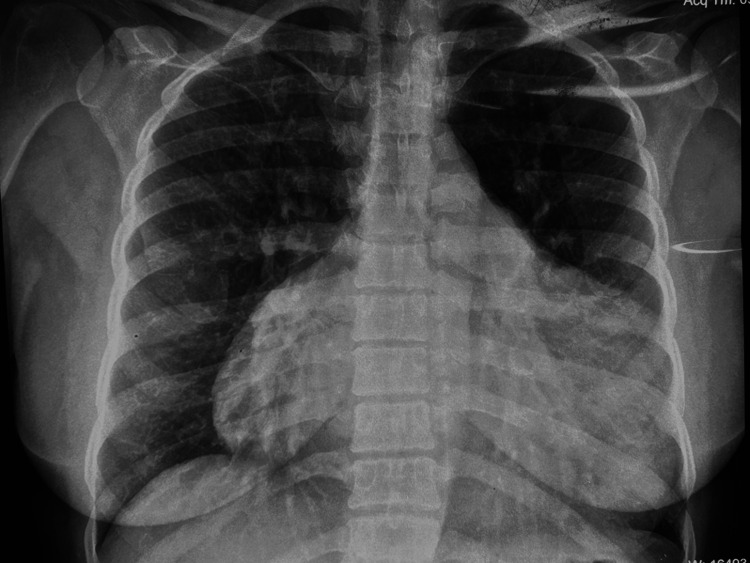
Chest radiograph (anteroposterior view) showing an enlarged cardiac silhouette (“water-bottle” sign of pericardial effusion).

On admission, the patient was afebrile, with a blood pressure of 110/70 mm Hg, heart rate of 125 beats/minute, respiratory rate of 30 breaths/minute, and oxygen saturation of 98% on room air. Jugular venous pressure was elevated, and heart sounds were muffled. There was no pedal edema, hepatomegaly, or pulsus paradoxus. The electrocardiogram demonstrated right bundle branch block with right-axis deviation. Transthoracic echocardiography revealed a large circumferential pericardial effusion with right atrial diastolic collapse, preserved left ventricular systolic function, and a 23-mm ostium secundum ASD with left-to-right shunt, consistent with impending pericardial tamponade. Chest radiograph showed an enlarged cardiac silhouette and clear lung fields (Figure 1).

**Figure 2:**
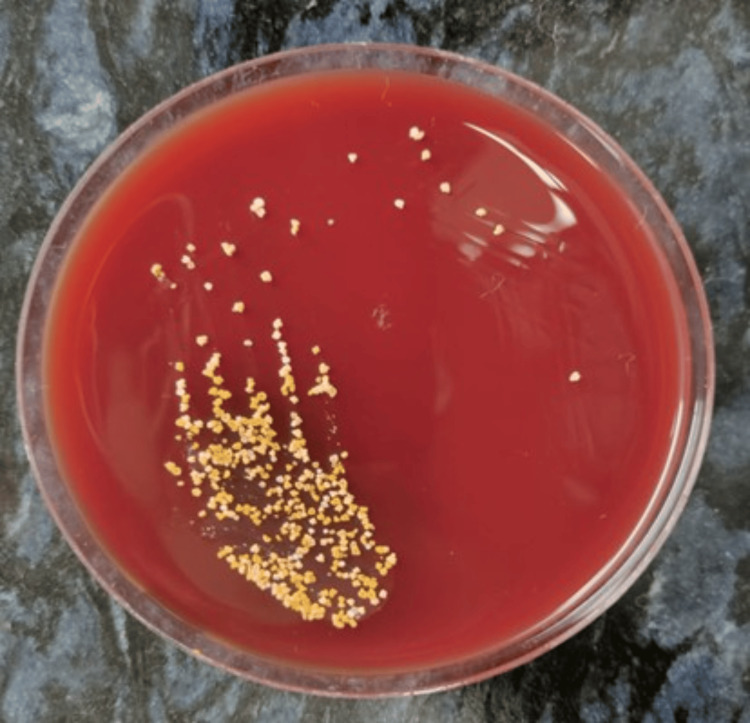
Blood agar plate showing dry, pitting colonies of Nocardia spp.

**Figure 3:**
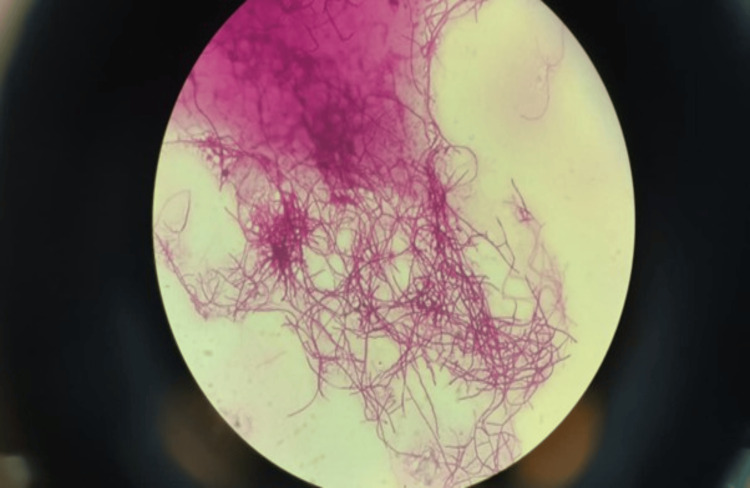
Thin, branching, filamentous acid-fast bacilli (magnification: 1,000×) grown on culture suggestive of Nocardia spp.

An emergency ultrasound-guided pericardiocentesis was performed, yielding approximately 650 mL of blood-tinged, exudative fluid, and a pigtail catheter was left in situ for drainage. Pericardial fluid analysis revealed an albumin of 2.7 g/dL (serum albumin: 3.8 g/dL), total protein of 5.5 g/dL (serum protein: 7.2 g/dL), lactate dehydrogenase (LDH) of 486 U/L (serum LDH: 526 U/L), glucose of 164 mg/dL (blood glucose: 182 mg/dL), and amylase of 42 U/L. Cytology did not show any evidence of malignancy. The total leukocyte count was 10,000/mm³ with 80% polymorphonuclear and 20% mononuclear cells. The patient was initially started on empiric antitubercular therapy (isoniazid 300 mg + rifampin 600 mg + pyrazinamide 1,500 mg + ethambutol 1,200 mg) once daily. Acid-fast bacilli stain, GeneXpert, and KOH mount were all negative, and adenosine deaminase (ADA) was 13 U/L (normal value <30 U/L). Gram staining showed filamentous, branching, gram-positive bacilli. Modified Ziehl-Neelsen staining showed weakly acid-fast bacilli. After seven days of incubation on blood agar, chalk-white, dry colonies appeared, confirming *Nocardia* spp. (Figures 2, 3). During this time, the patient continued to have low-grade fever with the pericardial catheter draining hemorrhagic fluid.

Species-level identification with matrix-assisted laser desorption/ionization with tandem time-of-flight analysis revealed Nocardia brasiliensis. The patient was immunocompetent. HIV enzyme-linked immunosorbent assay, anti-hepatitis C virus, and hepatitis B surface antigen were negative. HbA1c was 6.8%, thyroid-stimulating hormone was within the normal range, erythrocyte sedimentation rate (ESR) was 52 mm/hour, and total leukocyte count was normal. Based on the culture result, the diagnosis of nocardial pericarditis with tamponade was established. Infectious disease consultation advised discontinuation of antitubercular therapy and initiation of therapy with trimethoprim-sulfamethoxazole (TMP-SMX) (160 mg/800 mg two tablets twice daily) and linezolid 600 mg twice daily. Subsequently, antimicrobial susceptibility testing showed sensitivity to imipenem, linezolid, minocycline, amoxicillin-clavulanate, ceftriaxone, and ciprofloxacin, and resistance to TMP-SMX. The patient was continued on linezolid 600 mg twice daily along with amoxicillin-clavulanate (500 mg/125 mg one tablet thrice daily), with a planned duration of six months. A CT scan of the chest and abdomen showed subcentimetric mediastinal lymph nodes with no other focus of Nocardial infection. The patient’s dyspnea improved steadily over the next seven days, with no recurrence of effusion on repeat echocardiography. She remained afebrile, hemodynamically stable, and inflammatory markers normalized (ESR: 15 mm/hour). The pericardial catheter was subsequently removed once the drainage was less than 50 mL for two consecutive days, and the patient was discharged. Follow-up after three days of discharge showed clinical stability and no evidence of re-accumulation on echocardiography. At one-month review, she remained asymptomatic (New York Heart Association Class I) with normal left ventricular function and no pericardial collection. Long-term follow-up was planned to monitor for relapse or medication toxicity.

## Discussion

Nocardial pericarditis is an exceedingly rare entity, particularly in immunocompetent individuals, and poses a significant diagnostic challenge due to its nonspecific presentation and slow-growing nature, requiring selective culture media. Nocardial infections can be acquired through various routes, including inhalation, ingestion, and direct inoculation of the skin. *Nocardia* species can hematogenously disseminate and involve multiple organ systems, with pulmonary-limited infection reported in approximately 39% of cases, central nervous system involvement in 9%, cutaneous disease in 8%, and disseminated infections in 32%; pericardial involvement is very rare and can occasionally present as purulent effusions requiring drainage [4]. While *Nocardia* species leading to infective endocarditis in congenital heart disease is well-known, nocardial pericarditis in congenital heart disease has not been previously documented [5]. Among pathogenic species, *N. asteroides* accounts for the majority of respiratory and disseminated infections, whereas *N. farcinica* is more virulent, prone to antibiotic resistance, and has a higher propensity for dissemination [6].

Most nocardial infections occur in immunocompromised hosts, such as those with HIV infection, organ transplantation, or other conditions impairing cell-mediated immunity [7]. Diabetes may confer a subtle predisposition even when well-controlled, as in our patient [8]. Table 1 summarizes cases of *Nocardia* pericarditis reported in the literature in immunocompromised and immunocompetent patients [9-20]. Clinically, nocardial pericarditis can closely mimic tuberculous pericarditis, especially in endemic regions, with massive pericardial effusions, blood-tinged exudates, and elevated inflammatory markers [9]. Differentiating features include polymorphonuclear predominance in pericardial fluid, normal ADA levels, partial acid-fastness on modified Ziehl-Neelsen staining, and a negative GeneXpert, highlighting the importance of microbiological confirmation before initiating long-term empiric therapy [9]. Prompt pericardial drainage combined with prolonged targeted antibiotic therapy, typically TMP-SMX, is essential to prevent tamponade and ensure favorable outcomes. Alternative drugs, such as linezolid, meropenem, imipenem, amikacin, ceftriaxone, and amoxicillin-clavulanate, can also be used alone or in combination, based on local epidemiology and susceptibility profiles [8,9]. Our patient, an immunocompetent middle-aged woman with a congenital ASD, demonstrated gradual clinical improvement and near-complete resolution of pericardial effusion following drainage and linezolid and amoxicillin-clavulanate therapy, underscoring the need for high clinical suspicion for atypical organisms such as *Nocardia *even in patients without classic risk factors, as well as the value of early intervention and careful follow-up to ensure complete recovery.

**Table 1:** Cases of Nocardia pericarditis reported in the literature. TMP-SMX = trimethoprim-sulfamethoxazole; HIV = human immunodeficiency virus; AIDS = acquired immunodeficiency syndrome

Immunocompromised patients						
Age	Gender	Cardiac tamponade	Therapy	Outcome	Underlying condition	Reference
52	Male	No	IV TMP/SMX + ceftriaxone + amikacin oral amoxicillin/clavulanate + TMP/SMX	Recovered	HIV/AIDS	[9]
32	Male	No	IV TMP/SMX	Recovered	HIV/AIDS	[10]
34	Male	No	IV TMP/SMX	Died	HIV/AIDS	[10]
34	Male	Yes	IV TMP/SMX oral sulfadiazine	Recovered	HIV/AIDS	[11]
42	Male	No	IV TMP/SMX (allergic) + IV ceftriaxone + minocycline	Recovered	HIV/AIDS	[12]
24	Female	Yes	Oral TMP/SMX	Recovered	HIV/AIDS	[13]
44	Male	No	IV TMP/SMX + oral TMP/SMX	Recovered	HIV/AIDS	[14]
35	Female	Yes	IV TMP/SMX + ceftriaxone + doxycycline + oral TMP/SMX + doxycycline	Recovered	HIV/AIDS	[15]
37	Male	Yes	IV imipenem/cilastatin + TMP/SMX + oral TMP/SMX	Recovered	HIV/AIDS	[16]
32	Male	Yes	IV imipenem/cilastatin + TMP/SMX	Died	HIV/AIDS	[17]
Immunocompetent patients						
65	Female	No	IV imipenem/ceftriaxone/linezolid + oral TMP/SMX/doxycycline + oral TMP/SMX	Recovered	-	[18]
60	Male	Yes	Imipenem/cilastatin + linezolid + TMP/SMX	Recovered	Heavy alcohol use	[19]
63	Male	No	TMP/SMX	Recovered	-	[20]

## Conclusions

While tuberculosis remains the most common diagnosis for exudative effusions in tuberculosis-endemic countries, it must not be assumed without microbiological confirmation. *Nocardia *pericarditis can mimic tubercular pericarditis. Therefore, a high index of suspicion must be maintained for atypical microorganisms in the diagnosis of pericardial effusion. Early microbiological diagnosis and targeted antimicrobial therapy are the key to optimal patient response and outcomes.
